# A Cross-Sectional Study to Compare AI-Generated Educational Content Using Google Gemini for Medical Professionals With UpToDate on Pediatric Asthma

**DOI:** 10.7759/cureus.99286

**Published:** 2025-12-15

**Authors:** Anna Jijo, Maria Jijo, Swetha Manoj, Mira S Aridas, Chaitanya Kumar Javvaji

**Affiliations:** 1 Pediatrics, St. John's Medical College, Bangalore, IND; 2 Pediatrics, Apollo Institute of Medical Sciences and Research, Hyderabad, IND; 3 Pediatrics and Neonatology, Government Medical College, Kannur, Pariyaram, IND; 4 Neonatal and Developmental Medicine, Singapore General Hospital Outram Road, Singapore, SGP; 5 Pediatrics, Jawaharlal Nehru Medical College, Datta Meghe Institute of Higher Education and Research, Wardha, IND

**Keywords:** artificial intelligence, asthma, clinical decision support, educational content, google gemini, medical education, uptodate

## Abstract

Introduction: Accurate and up-to-date educational resources are crucial for medical professionals to deliver effective patient care, particularly in conditions like pediatric asthma, which has a high disease burden in children. Timely interventions are essential to manage this condition appropriately and to ensure better outcomes. With the rapid advancement of artificial intelligence in healthcare, AI tools like Google Gemini are being explored as quick and accessible alternatives for generating medical content.

Methods: A cross-sectional observational study was conducted to focus on four core topics related to the management of pediatric asthma. Prompts for each of the core topics were entered in Google Gemini and UpToDate to generate responses. The WebFx Readability Tool was used to assess readability utilizing metrics such as Flesch Reading Ease (FRE), Flesch-Kincaid Grade Level (FKGL), SMOG Index, word count, sentence count, words per sentence, difficult word count, and percentage. The collected data were analyzed using the Mann-Whitney U test, and a p-value of < 0.05 was considered statistically significant.

Results: When comparing the readability characteristics between UpToDate and Google Gemini, statistically significant differences were found, indicating that Google Gemini is more accessible for individuals with lower literacy skills. UpToDate received higher scores on the Simple Measure of Gobbledygook (SMOG) index across all four core topics, denoting it as hard to understand for the normal population. Google Gemini scored a greater difficulty word percentage across all four topics.

Conclusion: Google Gemini was found to use more complex vocabulary while still maintaining overall accessibility, making it appropriate for patients with lower literacy levels. Although certain readability parameters demonstrated Google Gemini to be a more reader-friendly tool for assessing and understanding medical content, the high percentage of difficult words may make it more challenging for younger individuals and lower socio-economic populations to access.

## Introduction

Asthma is a common chronic inflammatory disease of the airways that affects millions of children worldwide, with a particularly high burden in those under the age of 12 [1]. Management strategies for pediatric asthma have evolved over the years and are continuously updated in clinical guidelines to ensure timely treatment, control of symptoms, and prevention of exacerbations. For medical professionals, especially those in training or primary care, access to concise, comprehensible, and up-to-date clinical information is essential for effective decision-making.

Traditionally, evidence-based platforms such as UpToDate have been widely used by clinicians for real-time information. However, the increasing accessibility of generative artificial intelligence (AI) tools like Google Gemini presents a new modality for accessing clinical knowledge. While these tools can offer rapid responses, questions remain about the readability and complexity of their output, particularly when compared to professionally curated resources. Previous research has shown that readability levels in patient education materials are often above the recommended sixth-to-eighth grade level, which may be inappropriate for public use and even pose a cognitive burden for busy professionals [2].

The American Medical Association (AMA) and the National Institutes of Health (NIH) recommend that patient educational materials be written at a sixth-grade and eighth-grade reading level, respectively [3]. With increasing reliance on AI tools for preliminary medical education, it is essential to assess whether these tools provide content that is not only accurate but also appropriately pitched in terms of complexity for the intended audience.

The overarching research question for this study was to determine how the readability and linguistic complexity of pediatric asthma educational content generated by Google Gemini compares with that of UpToDate when used by medical professionals. The specific objectives were to evaluate readability using standard metrics such as FRE, FKGL, and SMOG Index; analyze structural characteristics, including word count, sentence count, words per sentence, difficult word count, and difficult word percentage; and determine whether these differences between the two sources are statistically significant.

## Materials and methods

This was a cross-sectional observational study conducted over a two-month period, from April to May 2025. The study design was chosen to allow for a structured comparison of textual outputs from two different information sources across a predefined set of clinical topics. Since the research did not involve human participants, patient data, or any direct interaction with individuals, it did not fall within the scope of studies requiring ethical oversight. Consequently, formal approval from the Institutional Ethics Committee was not sought. This approach is consistent with international research ethics guidelines for studies utilizing secondary data sources and publicly available content.

The focus of this study was on the management of pediatric asthma, given its high prevalence and the critical role of guideline-based care in improving outcomes. Four core topics were identified as being particularly relevant for clinical decision-making in children under 12 years of age. These topics were selected based on their representation in international pediatric asthma guidelines, their importance in everyday clinical practice, and their coverage in educational resources. The selected topics were (1) persistent asthma: controller therapies, which addresses long-term pharmacological management; (2) acute exacerbations: home and office management, focusing on early intervention in primary care or home settings; (3) acute symptom relief: quick-relief treatment, which covers the use of rescue medications; and (4) emergency department care: exacerbation management, detailing advanced interventions for severe presentations.

For each of the four selected topics, the exact phrase of the topic title was entered into the Google Gemini [4] large language model (LLM) interface, using the version available as of April 2025 and default settings. The verbatim prompts used were “Asthma in children younger than 12 years: Management of persistent asthma with controller therapies,” “Acute asthma exacerbations in children younger than 12 years: Overview of home/office management and severity assessment,” “Asthma in children younger than 12 years: Quick-relief (rescue) treatment for acute symptoms” and “Acute asthma exacerbations in children younger than 12 years: Emergency department management.” The first complete response generated by Gemini for each prompt was collected in full without any editing, paraphrasing, or omission to maintain content fidelity. Only one output was recorded per prompt to reflect typical user interaction.

In parallel, the same topics were searched in UpToDate [5] using a professional subscription login on 25 April 2025 to ensure access to complete and up-to-date content. The retrieved text was copied in full and transferred into separate digital files labelled according to topic and source, with only minor formatting elements (e.g., hyperlinks and headings) removed to avoid influencing readability metrics. All original wording and structure were retained.

Readability analysis was performed using the WebFX readability tool [6], which incorporates multiple standardized indices to assess the complexity and accessibility of written material. For each document, the following readability metrics were calculated: Flesch Reading Ease (FRE), which provides a numerical score indicating how easy the text is to read; Flesch-Kincaid Grade Level (FKGL), which estimates the U.S. school grade required to understand the text; and the SMOG Index, which predicts the years of education needed to comprehend the content. In addition, the total word count, total sentence count, average words per sentence, number of difficult words (as defined by the tool’s integrated dictionary), and the percentage of difficult words relative to the total word count were recorded. All measurements were conducted separately for each source and topic to allow direct comparison.

All extracted readability scores and text characteristics were entered into Microsoft Excel (Microsoft Corp., Redmond, WA, USA) for initial organization and verification. Statistical analysis was then conducted using IBM Corp. Released 2020. IBM SPSS Statistics for Windows, Version 26. Armonk, NY: IBM Corp. The Mann-Whitney U test was chosen as the primary statistical test to compare readability scores between Google Gemini and UpToDate outputs, as preliminary inspection of the data indicated a non-parametric distribution. Descriptive statistics were presented as medians with interquartile ranges. A two-tailed p-value of less than 0.05 was considered statistically significant for all comparisons. All tools (metrics, scoring systems) used in this study were publicly available and free to use.

## Results

The results reveal statistically significant differences in the complexity of outputs between the two platforms, raising important questions about their suitability for professional medical learning.

Table 1 presents the comparison of readability characteristics between UpToDate and Google Gemini. The Mann-Whitney U test was used to compare the distribution of responses generated by UpToDate and Google Gemini. There was no statistically significant difference in the word count (p= 0.200), sentence count (p= 0.486), Flesch Reading Ease score (p= 0.886), difficult word count (p= 0.486), or difficult word percentage (p= 0.057). However, based on the p-values obtained in Table 1, there is a statistically significant difference between the word/sentence count (p= 0.029), Flesch-Kincaid grade level (p= 0.029), and SMOG index (p= 0.029) generated by the two AI tools.

**Table 1:** Characteristics of responses generated by UpToDate and Google Gemini. +Mann-Whitney U test. P-values < 0.05 are considered statistically significant. [4,5]

	Median (IQR)		U Statistic	p-value^+^
	UpToDate	Google Gemini		
Word Count	1864.5 (828.7 – 7860.0)	796.0 (275.2 – 1955.7)	3	0.200
Sentence Count	78.5 (39.5 – 303.5)	61.5 (22.5 – 177.7)	5	0.486
Word/Sentence Count	23.7 (20.6 – 25.5)	12.1 (10.9 – 13.1)	0	0.029*
FRE	27.2 (9.1 – 31.7)	22.6 (21.0 – 30.9)	7	0.886
FKGL	15.3 (15.2 – 17.8)	13.2 (11.8 – 13.4)	0	0.029*
SMOG Index	13.3 (12.7 – 13.5)	10.9 (9.9 – 11.3)	0	0.029*
Difficult Word Count	383.5 (212.2 – 1640.0)	232.0 (80.7 – 507.7)	5	0.486
Difficult Word Percentage	21.8 (19.9 – 26.4)	29.2 (26.3 – 29.3)	1	0.057

**Figure 1:**
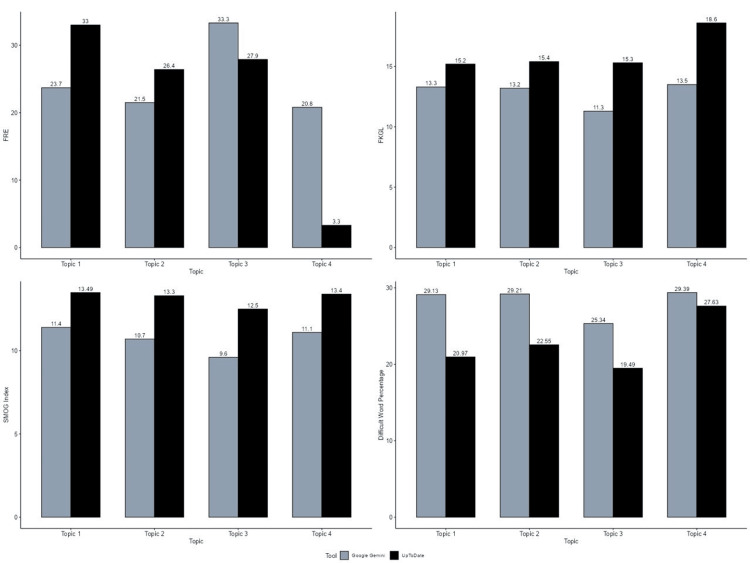
Shows the graphical representation of the comparison between FRE, FKGL, SMOG Index, and Difficult Word Percentage for the patient education guide generated by UpToDate and Google Gemini. FRE: Flesch Reading Ease, FKGL: Flesch-Kincaid Grade Level, SMOG: Simple Measure of Gobbledygook [4,5]

Figure 1 provides a graphical comparison of readability metrics, Flesch Reading Ease (FRE), Flesch-Kincaid Grade Level (FKGL), SMOG Index, and Difficult Word Percentage, content generated by UpToDate and Google Gemini across four topics related to pediatric asthma. Figure 1 demonstrates a consistent increase in FKGL and SMOG Index values for content generated by UpToDate as compared to Google Gemini across all four topics.

With respect to the FRE score, indicating clarity of text, Google Gemini scored lower than UpToDate in Topic 1 (23.7 vs. 33.0) and Topic 2 (21.5 vs. 26.4). Conversely, Google Gemini outperformed UpToDate in terms of FRE score in Topic 3 (33.3 vs. 27.9) and more significantly in Topic 4 (20.8 vs. 3.3). UpToDate generated output with a higher grade level than Google Gemini across all four topics on pediatric asthma. For example, in Topic 4, the Flesch-Kincaid Grade Level for Google Gemini is 13.5 compared to 18.6 for UpToDate, indicating that responses generated by Google Gemini may be more suitable for patients with lower literacy levels.

Similarly, in terms of the SMOG Index, UpToDate consistently demonstrated higher scores in comparison to the Google Gemini curated responses. For example, Topic 3 has an SMOG score of 9.6 for Google Gemini and 12.5 for UpToDate, suggesting that Google Gemini provides information that is more readily understood by the general population. A significant observation is that Google Gemini’s content scored a higher percentage across all four topics in terms of difficult word percentage. For instance, in Topic 2, Google Gemini had 29.21% difficult words versus 22.55% for UpToDate, which suggests a greater presence of complex vocabulary.

## Discussion

There is a statistically significant increase in the median word/sentence count, FKGL, and SMOG Index of UpToDate-generated content compared to that of Google Gemini.

AI allows educators to prepare future healthcare professionals to gain medical knowledge and improve their clinical skills. It fosters a more engaging and interactive environment, where students can easily understand complex topics of modern medicine [7]. Integrating AI into healthcare has allowed clinicians to optimize the way they treat their patients with regard to diagnosis, clinical testing, and treatment. As AI leverages large amounts of data in short amounts of time, it can surpass human performance and cut down on the time required for clinical decision-making [8].

Comprehension of a particular text is measured by its readability score, which is an important factor in determining whether the patient understands the educational material given to them. Better readability of educational materials is important with regard to physician-patient relationships, as well as with time constraints for healthcare professionals in acute settings [9]. Common metrics used to measure readability include the Flesch Kincaid Ease (FRE) score, the Flesch Kincaid Grade Level (FKGL), and the Simple Measure of Gobbledygook (SMOG) Index. Higher FRE scores indicate higher readability [10].

‌In this study, Google Gemini showed significantly better readability metrics than UpToDate, suggesting easier comprehension, although both showed content that remained complex overall, indicated by low FRE scores. A study by Karnan et al. (2024) comparing Google Gemini and ChatGPT-generated patient education guides showed similar patterns in sentence structure, language complexity, and moderate readability between the two AI tools [11]. A similar study by Swisher et al. (2024) demonstrated that ChatGPT could be used to revise and increase the readability of patient handouts, showing its capability as a tool in refining educational content [12]. These findings are similar to another study by Adithya et al. (2024), which evaluated AI-generated brochures for common emergency conditions, in which Gemini-generated content had higher readability than ChatGPT but similar sentence structure and length. This study also emphasizes that AI tools perform consistently across different topics [13].

While this study found that AI-generated content from Google Gemini offered better readability than resources like UpToDate, other studies have reported different outcomes. For example, a study by Lee et al. (2023) using ChatGPT in research related to anesthesiology demonstrated that the content generated lacked clinical depth and brought up additional issues of plagiarism in medical writing [14]. Similarly, a study by Sallam et al. (2023) on the use of ChatGPT in healthcare education demonstrated the additional difficulties of risk of bias, inaccurate or superficial content, and the lack of transparency regarding content generation [15]. As highlighted by Chen et al. (2023), discrepancies in this study and others can be attributed to the difference in the model of the AI tool, the prompt, or the topic of study chosen. Clear reporting of this information, along with multi-reviewer evaluation, will help to minimize variability [16].

Limitations

This study is limited by its small scope, as only one AI tool (Google Gemini) and a single clinical topic were evaluated across four subtopics, reducing statistical power and generalizability. As only one AI-generated response per prompt was analyzed, and outputs may vary with different tool versions or future updates, reproducibility may be affected. Although minor formatting elements were removed, differences in response length between Google Gemini and UpToDate may have introduced bias. Additionally, the study focused solely on readability and linguistic structure without assessing clinical accuracy, depth, or actual comprehension. Further research involving multiple AI tools, broader topics, and evaluation of both readability and content accuracy is recommended.

## Conclusions

This study compared AI-generated content from Google Gemini with UpToDate for pediatric asthma education and found that Gemini produced text with lower FKGL and SMOG Index values and shorter sentence structure, suggesting potentially easier readability. However, FRE scores varied across topics, indicating that readability was not uniformly superior. Despite the presence of more complex vocabulary, Gemini’s output may support quicker preliminary understanding.

These findings should be interpreted with caution, as only readability was assessed; content accuracy, clinical depth, and usability were not evaluated. Given the limited scope and potential variability in AI-generated content, further research involving broader topics, multiple AI models, and accuracy assessment is needed before incorporating AI tools into medical education or practice.
